# Feature fusion ensemble classification approach for epileptic seizure prediction using electroencephalographic bio-signals

**DOI:** 10.3389/fmed.2025.1566870

**Published:** 2025-08-04

**Authors:** Yazeed Alkhrijah, Shehzad Khalid, Syed Muhammad Usman, Amina Jameel, Muhammad Zubair, Haya Aldossary, Aamir Anwar, Saad Arif

**Affiliations:** ^1^Department of Electrical Engineering, Imam Mohammad Ibn Saud Islamic University (IMSIU), Riyadh, Saudi Arabia; ^2^King Salman Center for Disability Research (KSCDR), Riyadh, Saudi Arabia; ^3^Computer and Information Sciences Research Center (CISRC), Imam Mohammad Ibn Saud Islamic University, Riyadh, Saudi Arabia; ^4^Department of Computer Engineering, Bahria University, Islamabad, Pakistan; ^5^Department of Computer Science, Bahria University, Islamabad, Pakistan; ^6^Interdisciplinary Research Center for Finance and Digital Economy, King Fahd University of Petroleum and Minerals, Dhahran, Saudi Arabia; ^7^Computer Science Department, College of Science and Humanities, Imam Abdulrahman Bin Faisal University, Jubail, Saudi Arabia; ^8^School of Computing, University of Portsmouth, London Campus, London, United Kingdom; ^9^Department of Mechanical Engineering, College of Engineering, King Faisal University, Al Ahsa, Saudi Arabia

**Keywords:** AI in healthcare, epilepsy, electroencephalogram, epileptic seizure prediction, signal quality index, optimal spatial filter, 1DCNN, ensemble classifier

## Abstract

**Introduction:**

Epilepsy is a neurological disorder in which patients experience recurrent seizures, with the frequency of occurrence more than twice a day, which highly affects a patient's life. In recent years, multiple researchers have proposed multiple machine learning and deep learning-based methods to predict the onset of seizures using electroencephalogram (EEG) signals before they occur; however, robust preprocessing to mitigate the effect of noise, channel selection to reduce dimensionality, and feature extraction remain challenges in accurate prediction.

**Methods:**

This study proposes a novel method for accurately predicting epileptic seizures. In the first step, a Butterworth filter is applied, followed by a wavelet and a Fourier transform for the denoising of EEG signals. A non-overlapping window of 15 s is selected to segment the EEG signals, and an optimal spatial filter is applied to reduce the dimensionality. Handcrafted features, including both time and frequency domains, have been extracted and concatenated with the customized one-dimensional convolutional neural network-based features to form a comprehensive feature vector. It is then fed into three classifiers, including support vector machines, random forest, and long short-term memory (LSTM) units. The output of these classifiers is then fed into the model-agnostic meta learner ensemble classifier with LSTM as the base classifier for the final prediction of interictal and preictal states.

**Results:**

The proposed methodology is trained and tested on the publicly available CHB-MIT dataset while achieving 99.34% sensitivity, 98.67% specificity, and a false positive alarm rate of 0.039.

**Discussion:**

The proposed method not only outperforms the existing methods in terms of sensitivity and specificity but is also computationally efficient, making it suitable for real-time epileptic seizure prediction systems.

## 1 Introduction

**Figure 1:**
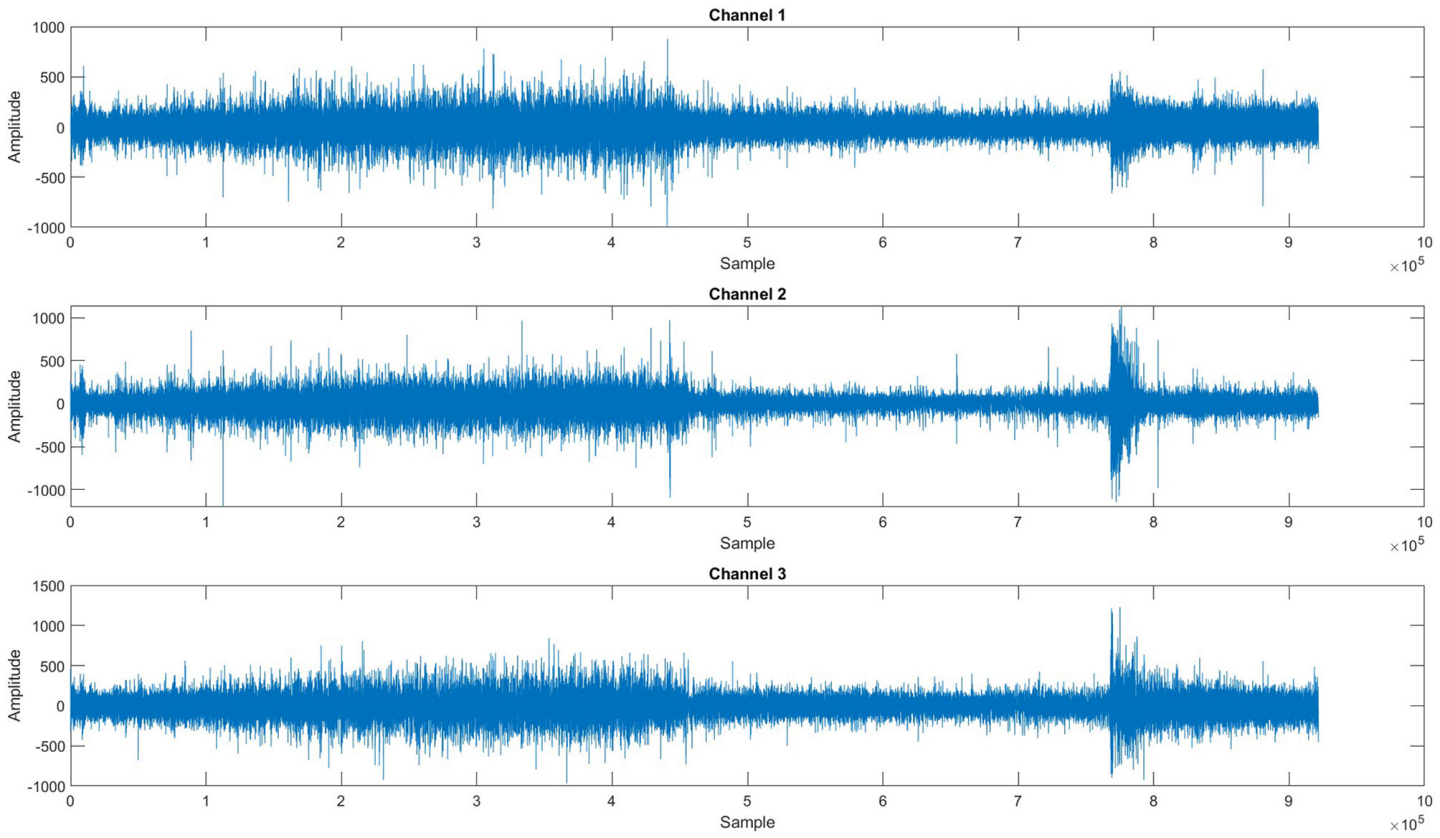
One-hour span session of EEG recordings for three channels.

Epilepsy is a neurological disorder in which patients suffer from seizures, and it affects their quality of life as a sudden seizure may cause an accident or injury while driving, climbing stairs, or walking on the road, etc. Seizure disturbs the activity of the brain, which can be observed by visualizing the electroencephalographic (EEG) signals recorded by placing electrodes on the scalp of the patient's brain (1). Seizures are divided into four states: interictal, the normal state; preictal, which starts a few minutes before the onset of seizure and ends with the seizure onset; ictal, in which the seizure occurs; and postictal, which starts after the seizure. Seizures can be categorized into two types, i.e., focal and generalized seizures. Focal seizures are normally treatable with surgical procedures, whereas generalized seizures can only be treated with the help of medicines; however, it has been observed that in 70% of the cases these seizures cannot be completely controlled with the help of medicines (2). Researchers (3–19) have proposed multiple methods to predict the onset of seizures before they occur by predicting the preictal state; however, accurate prediction remains a challenge due to multiple factors. EEG signals are susceptible to noise added during signal acquisition, high dimensionality due to the number of channels, and computational complexity of feature extraction and accurate classification. Figure 1 shows a plot of three EEG signals from 1-h continuous recordings. Accurate seizure prediction significantly impacts patient safety and quality of life by reducing the risks of sudden accidents or injuries during seizures. Despite advancements, clinicians and patients still face considerable challenges due to inaccurate seizure forecasting, leading to compromised safety and anxiety among epilepsy patients.

A typical method of epileptic seizure prediction involves preprocessing of EEG signals for noise removal and channel selection, followed by feature extraction and classification. Numerous techniques to preprocess EEG signals have been proposed in recent years for removing noise and artifacts such as eye blinks, eye movements, and muscle activity before feeding the data into the model. Fei et al. (6) and Usman et al. (14) proposed bandpass filters to preprocess the EEG signals. Wang et al. (20) has employed an infinite impulse response (IIR) bandpass filter and filtered the segmented data to filter out artifacts. Cho et al. (8) has used the fast Fourier transform (FFT). Common spatial pattern (CSP) is applied to reduce the effect of artifacts from EEG signals by Birjandtalab et al. (4). Researchers (14, 21, 22) have made use of the short-time Fourier transform (STFT) for preprocessing. Jana et al. (9) has utilized a pool-based technique with a 30-s window for noise reduction.

Duun-Henriksen et al. (23) selected channels based on the maximum variance, the difference in variance, and entropy. Entropy indicates the extent of disorder, impurity, and uncertainty, so the channels with the highest entropy were selected. To select channels that carry the highest information and are optimal, Daoud and Bayoumi (10) has selected channels with the maximum variance entropy product. Birjandtalab et al. (4) has used a random decision forest for channel selection. Cogan et al. (7) selected the best channel by ranking all the features based on the information gain for each subject. Parvez and Paul (24) checked the significance of each channel individually, then eliminated the channel of low significance and selected the best channels by calculating the average classification accuracy iteratively. Wang et al. (20) in their research study calculated a signal quality index (SQI), based on signal complexity. They brought three types of signals into consideration, and the optimal channels were selected accordingly.

Commonly used feature extraction methods include continuous wavelet transform (CWT), discrete cosine transformation (DCT), and discrete wavelet transform (DWT). Tsiouris et al. (25), Jana and Mukherjee (16), Alotaiby et al. (5), and Arif et al. (21) applied DWT to extract time-frequency features and then support vector machines (SVM) for predictions. Asharindavida et al. (11) utilized empirical mode decomposition (EMD) for feature extraction. Birjandtalab et al. (4), Birjandtalab et al. (3), and Borhade et al. (12) employed power spectral density (PSD) for feature extraction. Fei et al. (6) has applied a FrFT-based chaos method to obtain relevant features. Both time and frequency domain features, along with total energy spectrum and energy percentage-based features, were extracted to be used as input to the classifier (15). Zhang et al. (13) has made use of CSP-based feature extraction. Truong et al. (22) and Arif et al. (26) used STFT to extract features. Deep learning (DL) can also be used for feature extraction, as Daoud and Bayoumi (10) has extracted features through DL techniques.

Once features are extracted, the next task is to distinguish the signal between interictal and preictal states. Researchers have made use of machine learning (ML) and DL classifiers for the classification of EEG signals in seizure prediction methods. SVM with cross-validation was used for classification by Tamanna et al. (15), Alotaiby et al. (5), and Asharindavida et al. (11), a least square SVM classifier was applied to classify the EEG signals. Back-forward propagation neural networks (BPNN) and linear discriminant analysis (LDA) were also used for classification (6, 11, 13). Fei et al. (6), Usman et al. (14), Alotaiby et al., (5), Asharindavida et al. (11), and Alickovic et al. (27) employed k-nearest neighbor (kNN), and random forest (RF) for classification. In the study by Truong et al. (22), a convolutional neural network (CNN) was utilized for the classification of preictal and interictal states. Daoud and Bayoumi (10) and Alotaiby et al. (5) have used DL models [multilayer perceptron (MLP), deep CNN (DCNN), bidirectional LSTM (Bi-LSTM)] for classification tasks.

DL and EEG-based seizure prediction has advanced significantly in recent years. By successfully modeling EEG data across several spatial and temporal scales, Dong et al. (28) proposed a novel multi-scale spatio-temporal attention network (MSAN), which increased the accuracy of seizure prediction. Alasiry et al. (29) suggested a heterogeneous graph neural network (GNN) that enhanced clinical interpretability and predictive performance by capturing intricate EEG channel interactions. A CNN-Bi-LSTM hybrid model was presented by Cao et al. (30), who also developed a feature-level fusion technique that showed improved performance for epileptic seizure prediction across multiple datasets. Bi-LSTM consistently outperformed other recurrent neural network (RNN) structures like gated recurrent units (GRU), MLP, and DCNN for seizure prediction tasks according to an ablation study conducted by Bajaj and Sharma (31) on a variety of LSTM-based architectures. A novel mobile network information gain (M-NIG) technique was presented by Meng et al. (32) with a focus on individual-specific multi-channel EEG networks to lower noise and greatly improve prediction robustness. Notwithstanding these developments, there are still issues that need to be addressed, mainly in the areas of computational complexity, practicality for real-time clinical applications, efficient dimensionality reduction, and reliable handling of class-imbalanced data. These issues together highlight the necessity for further research.

Current approaches for epileptic seizure prediction predominantly utilize all available EEG channels. This practice is computationally expensive, increases time complexity, and raises hardware and financial costs, highlighting the need for methods that can identify and utilize only the most informative channels. The high dimensionality of EEG data often affects the efficiency and accuracy of predictive models. Despite its critical impact, this challenge has been largely overlooked in existing studies, necessitating effective dimensionality reduction techniques to enhance prediction performance. Many researchers have not adequately addressed the issue of class imbalance, a prevalent challenge in seizure prediction where certain classes (e.g., seizure events) are underrepresented compared to others. This imbalance can skew model performance and compromise prediction reliability.

We propose a novel method for epileptic seizure prediction to address these research gaps, which have been identified after a comprehensive literature review. In the first step, the Butterworth filter is applied, followed by wavelet and Fourier transforms for denoising of EEG signals. A non-overlapping window of 15 s is selected to segment the EEG signals, and an optimal spatial filter is applied to reduce the dimensionality. Handcrafted features, including both time and frequency domains, have been extracted and concatenated with the customized one-dimensional CNN (1DCNN)-based features to form a comprehensive feature vector. It is then fed into three classifiers, including SVM, RF, and LSTM units, and the output of these classifiers is then fed into a model-agnostic meta learner (MAML) ensemble classifier with LSTM as base classifier for the final prediction of interictal and preictal states. The contributions of this research include:

- Introduced a novel technique to identify the most informative EEG channels, improving prediction accuracy while significantly reducing computational costs, a key challenge in real-time applications.
- Developed an effective dimensionality reduction method to deal with the high-dimensional nature of EEG data, which affects the performance of prediction algorithms.
- Proposed a surrogate channel by combining optimal EEG channels that contribute the most to seizure prediction.
- Demonstrated the effectiveness of the proposed method on the publicly available CHB-MIT dataset, achieving a sensitivity of 99.34% and specificity of 98.67% with a false positive alarm rate of 0.03. These results outperform various state-of-the-art techniques, establishing a new benchmark in epileptic seizure prediction.

## 2 Methodology

**Figure 2:**
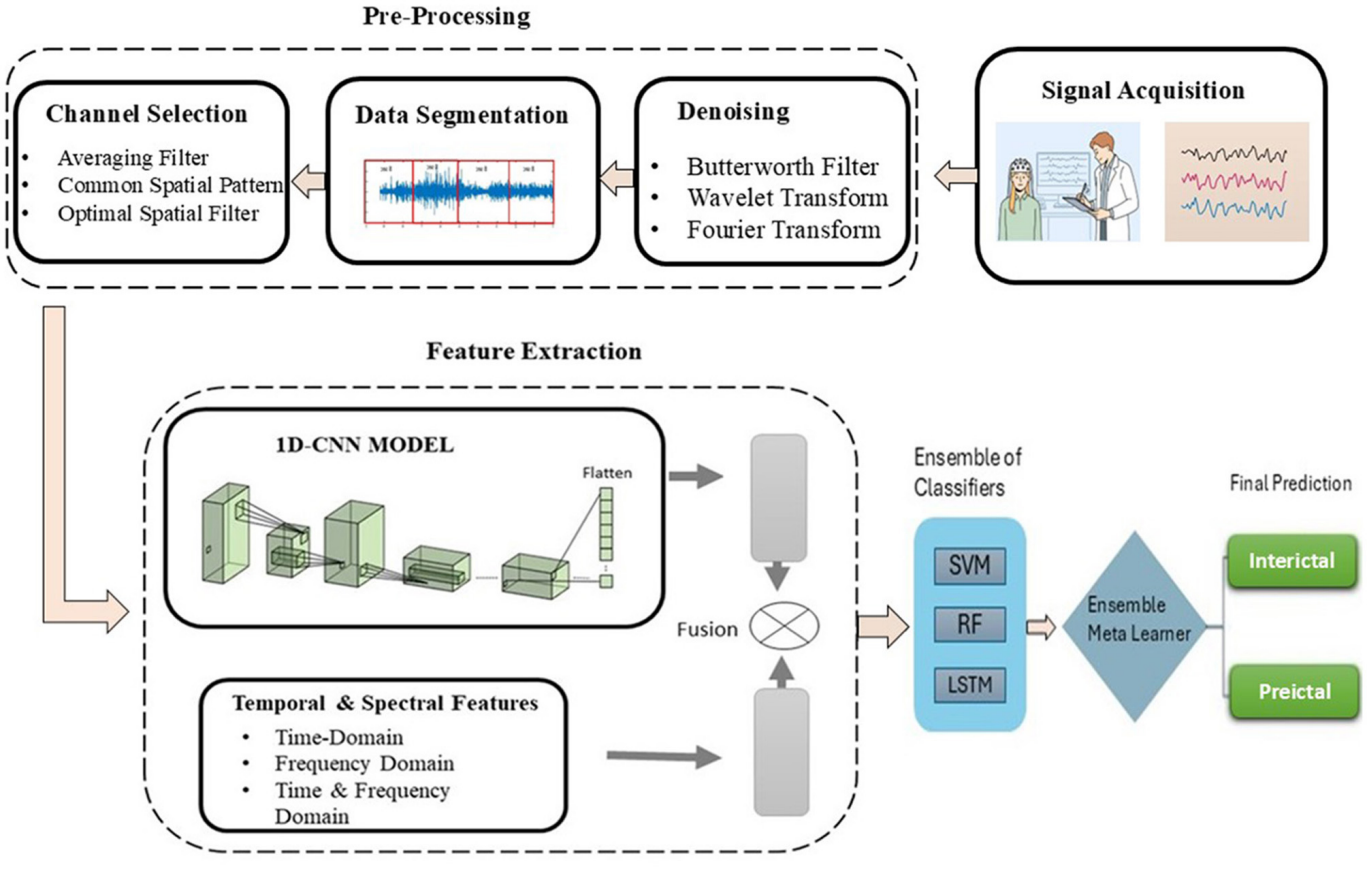
Flow diagram of the proposed methodology of epileptic seizure prediction.

To overcome the identified limitations and enhance seizure prediction accuracy, our methodology strategically targets the three main challenges: noise reduction in EEG signals, dimensionality reduction, and class imbalance mitigation. We propose a novel method of epileptic seizure prediction using EEG signals. It consists of three steps, including the preprocessing of EEG signals, feature extraction, and classification between preictal and interictal states. The preprocessing step involves segmentation of EEG signals into equal-size segments using a non-overlapping window, followed by multistage noise removal using Butterworth filter, wavelet, and Fourier transforms, and conversion of multi-channel EEG signals into a single surrogate channel. After preprocessing, both handcrafted and automated features have been extracted and concatenated to form a single feature vector. Time and frequency domain features include statistical and spectral signatures, whereas a customized architecture of 1DCNN has been proposed to extract automated features. Figure 2 shows the flow diagram of the proposed method. The following subsection presents all three steps of the proposed methodology in detail.

### 2.1 Preprocessing of EEG signals

**Figure 3:**
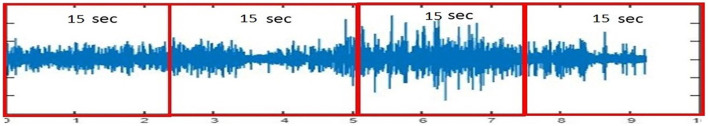
EEG data segmented into 15-s windows.

Due to the inherent susceptibility of EEG signals to noise from artifacts and external sources, a robust preprocessing strategy is critical to ensure data quality for reliable seizure prediction. In this research, we used a publicly available CHB-MIT dataset (33) that comprises EEG recordings of 24 pediatric individuals recorded in the Children's Hospital Boston. The dataset has been annotated by the medical experts with the start and end time of the seizure for each session of all individuals. EEG signals have been recorded with 23 channels and follow the 10–20 electrode placement method. The dataset has been sampled at 256 Hz and totals 644 h of recordings. We have divided EEG signals into equal-sized segments with the help of an equal-sized, non-overlapping window of 15 s. Figure 3 shows the plot of segmented EEG signals proposed in this research.

After segmenting the EEG signals, preictal and interictal signals were separated. Preictal and interictal samples were carefully selected, considering that preictal and postictal samples may overlap. Therefore, we included only those sessions for interictal state samples where no seizure onset occurred within two sessions before or after. Preictal state has been considered as 30 min before the onset of the seizure, provided that there was no seizure in the last session to avoid the postictal state overlapping with the preictal state. EEG signals are sensitive to noise, making it essential to apply various techniques to remove noise and artifacts, ensuring that the raw data is suitable for further processing. Methods include: Butterworth bandpass filter, EMD, FFT, CWT, DWT, and CSP, which help deal with noise and artifacts. Additionally, a window duration, overlapping and non-overlapping, can also be used to reduce the effect of noise to achieve better results.

**Figure 4:**
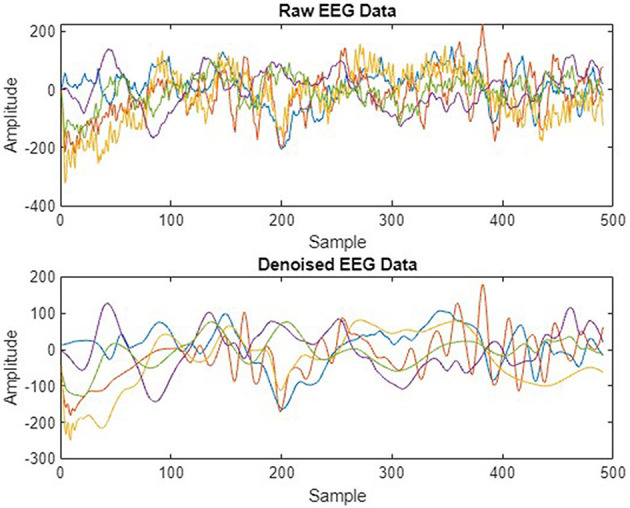
Raw vs. denoised EEG signals.

**Figure 5:**
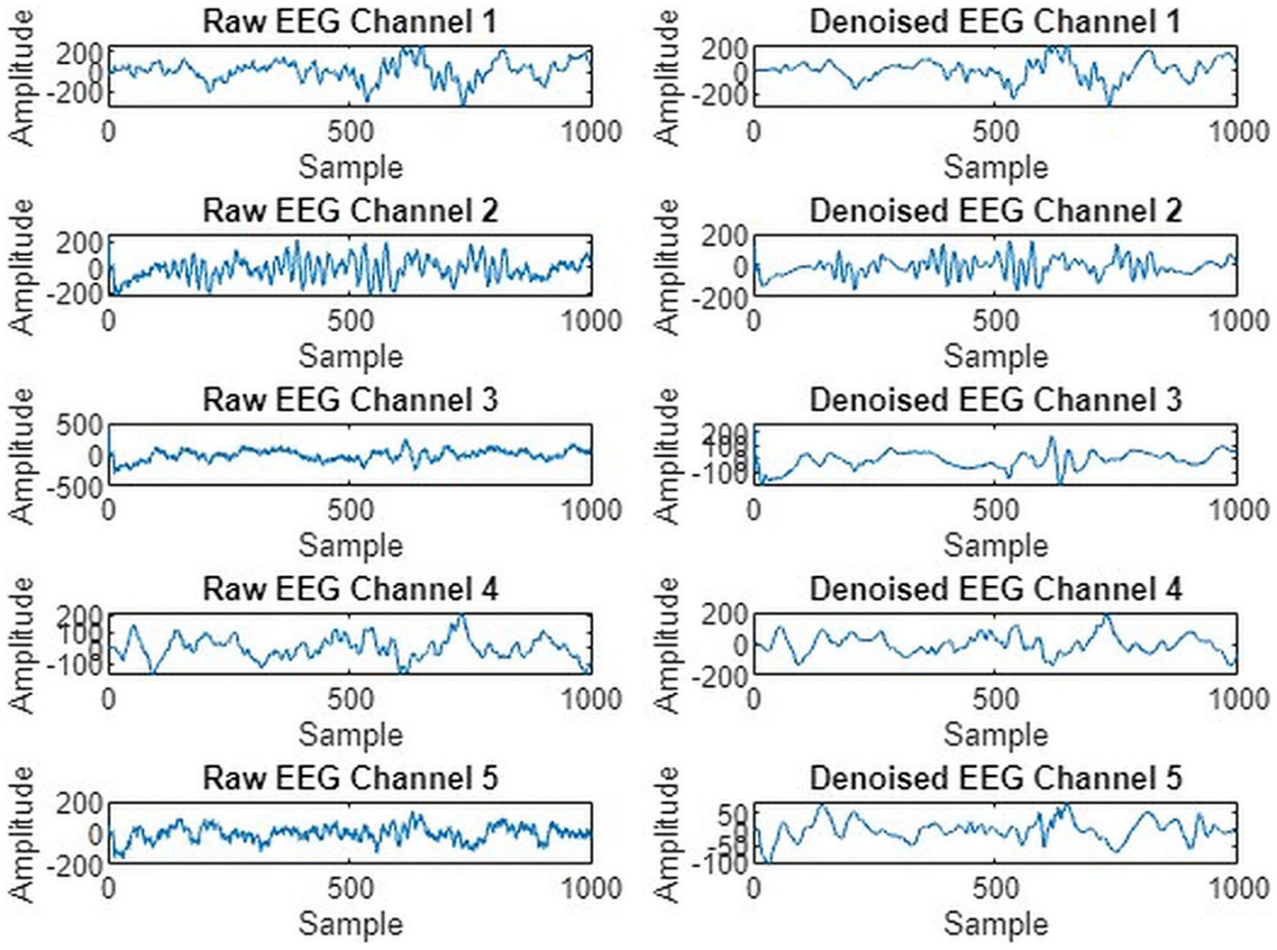
Five EEG channel waveforms before and after noise removal.

We preprocessed EEG signals to remove noise and artifacts to enhance signal quality, as shown in Figure 4. The wavelet transform and Butterworth filter, a high-pass filter with a cutoff frequency of 0.5 Hz and a low-pass filter with a cutoff frequency of 40 Hz, were applied. These filters were used to remove low-frequency, high-frequency drifts and fluctuations caused by internal and external sources during data recording. Figure 5 illustrates the raw signal alongside the denoised signals after applying these filters. The EEG signals are acquired through multi-channel recordings. Using a large set of channels leads to computational complexity. Additionally, not all channels provide valuable insights for seizure prediction. The use of all channels can also result in misclassifications of seizures. To address these issues, channel selection is a critical step in reducing the number of channels while preserving essential information.

**Figure 6:**
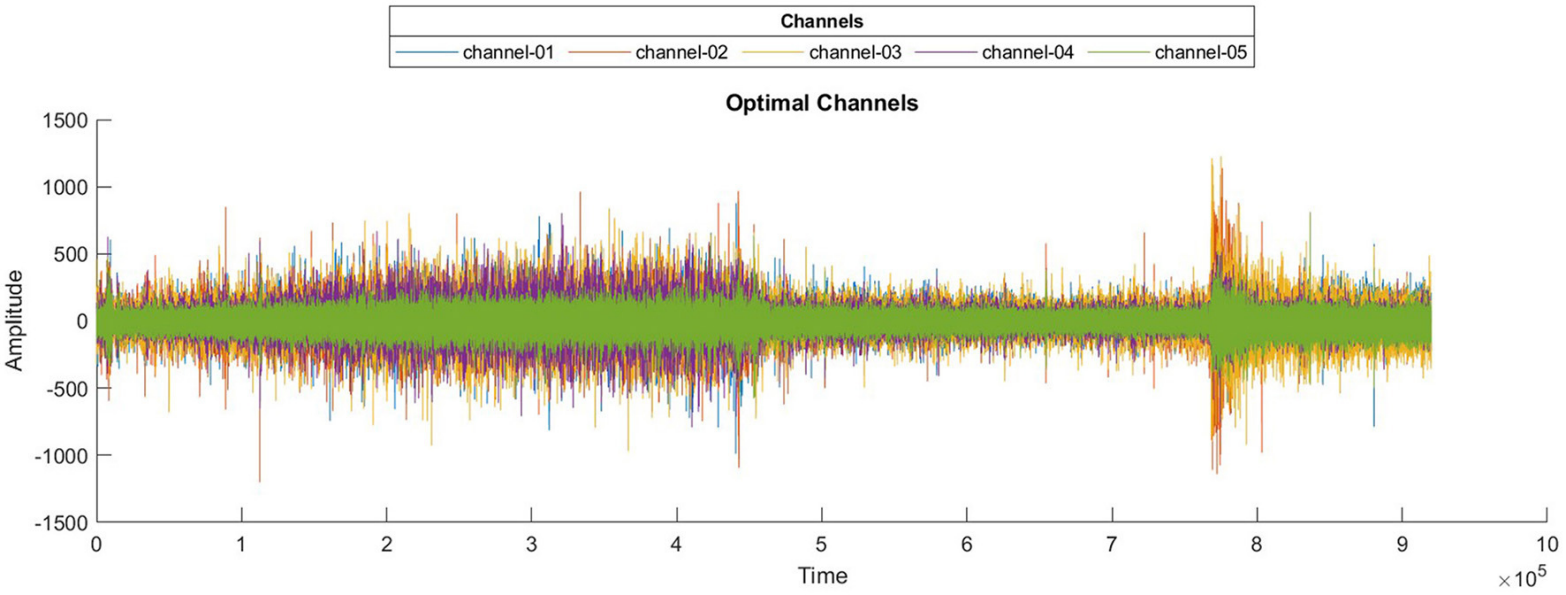
Waveforms of selected optimal EEG channels.

The number of channels is not only reduced, but optimal channels are also combined, which are highly contributing to seizure prediction, to make a surrogate channel. The channels are selected based on two criteria: high SQI and maximum variance. A higher SQI indicates superior signal quality, while lower values suggest poorer quality. Higher variance suggests increased brain activity. By selecting channels that meet these criteria, we ensure that the most informative and relevant channels are retained, leading to more accurate and efficient seizure prediction. A combined plot of all five selected channels is presented in Figure 6.


(1) $$\begin{array}{l} V_{ict}\left(C\right)=\frac{1}{k}\overset{k}{\underset{i=1}{\sum }}\left(x_{c}\left(i\right)-\mu_{c}\right){)}^{2} \end{array}$$

(2) $$\begin{array}{l} Selected\;Channel=\underset{1:N}{\max}\left\{Vict\left(c\right)\right\} \end{array}$$

#### 2.1.1 Surrogate channel

Given the computational inefficiency caused by analyzing high-dimensional EEG data from multiple channels, we introduce a surrogate channel technique. Unlike previous methods that typically analyze all channels equally, our approach identifies and combines the most informative EEG channels into a single surrogate channel, significantly reducing computational complexity while maintaining prediction accuracy. High-dimensional EEG signals pose significant problems in EEG analysis, including increased computational cost and a higher risk of overfitting to noise rather than extracting meaningful patterns. Addressing this issue can not only increase the performance of the classifier but also reduce the computational complexity. To convert multiple EEG channels into a surrogate channel, an averaging filter, CSP, and an optimal spatial filter were applied. These techniques were applied to increase the signal-to-noise ratio (SNR) and variance interval between two classes. The averaging filter is a method used to increase the SNR by replacing each sample with the average value of neighboring samples within a defined window. This averaging filter calculates the mean of all the channels to form a single channel (surrogate channel). The surrogate channel obtained after applying an averaging filter contains more SNR than multiple channels. The surrogate channel aims to capture the collective signal from multiple electrodes, potentially improving interpretability and simplifying analysis.

Despite its effectiveness in noise reduction, residual noise may persist in the surrogate channel, necessitating further refinement or the consideration of complementary filtering techniques to optimize signal quality for further analysis. The CSP filter is a technique that is frequently used in EEG signal processing to enhance the discriminative features of EEG signals by spatially filtering them. The CSP algorithm identifies spatial filters that increase the variance of EEG signals for one class while minimizing it for another class. CSP not only increases the SNR but also enhances the variance interval between two or more classes. This suggests that relevant information becomes more distinct while noise is effectively suppressed. In essence, CSP can convert a multi-channel EEG signal into a surrogate channel that encapsulates the most discriminative features for the task at hand.

#### 2.1.2 Mitigating the class imbalance problem

Class imbalance is a critical challenge in EEG-based seizure prediction because the number of preictal segments (indicating impending seizures) is significantly fewer than interictal segments (non-seizure states), potentially biasing prediction models. To address this imbalance, we utilize advanced oversampling techniques. Imbalanced data refers to too many instances in one class and too few examples in another. Imbalanced data can highly affect the model's overall effectiveness and make it difficult for the model to distinguish between the decision boundaries of different classes. One of the solutions to deal with this is to over-sample the instances in the minority class. Over-sampling can be attained by simply duplicating instances from the minority class in the training dataset before fitting a model. This does not give any extra information to the model, but it can deal with the data imbalance issue. An enhancement on duplicating instances from the minority class is to synthesize new instances from the minority class. In this study, data splitting was performed after an initial oversampling process to address class imbalance and improve model performance. Specifically, we utilized the synthetic minority over-sampling technique (SMOTE) and the soft prototype instance discrimination for enhancing representation (SPIDER) techniques to generate additional synthetic samples and improve the representation of minority classes. SMOTE selects a minority class instance randomly and then finds its *k* nearest minority class neighbors.

**Figure 7:**
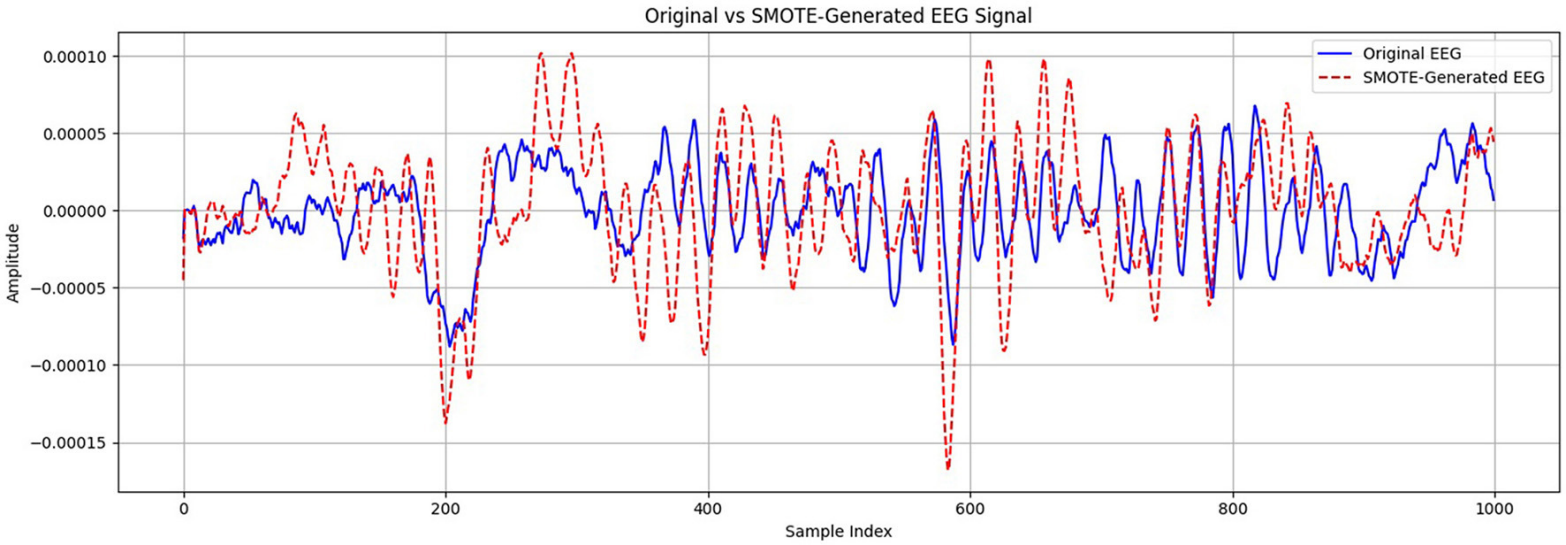
Comparison of original and SMOTE-generated EEG signals for the minority class.

The synthetic instances are then generated as a convex combination of the selected instances. SPIDER works by producing synthetic samples for the minority class in accordance with prototype instances. Prototype instances are representative samples from the minority class that capture its characteristics. SPIDER synthesizes new instances by perturbing these prototypes, creating variations that are still representative of the minority class. After applying these oversampling methods, the dataset was partitioned into training and validation subsets. Figure 7 presents a visual comparison between an original EEG segment and a synthetic sample generated using the SMOTE. The synthetic EEG maintains the temporal rhythm and amplitude range of the original signal, with minor variations that reflect the data-driven interpolation characteristics of SMOTE. To assess the fidelity of the generated samples, we evaluated similarity using statistical metrics such as Pearson correlation and dynamic time warping (DTW), both of which confirmed a high degree of alignment between the original and synthetic signals. This validates the suitability of SMOTE for augmenting the minority class in EEG-based classification tasks without introducing unrealistic distortions.

### 2.2 Feature extraction from EEG signals

Effective feature extraction is crucial to distinguish between seizure states clearly. Thus, we combine handcrafted temporal and spectral features with automated DL-based features to ensure high inter-class separability, which is key for robust classification. After preprocessing and channel selection, feature extraction is a critical step in the prediction of epileptic seizures. To capture both interpretable signal characteristics and complex spatial-temporal dependencies, we adopted a hybrid feature extraction strategy. Handcrafted features such as Hjorth parameters and entropy measures are well-established in EEG analysis for their ability to reflect signal complexity and variance.

#### 2.2.1 Handcrafted features

Various techniques for feature extraction are presented in the literature, including both handcrafted and automatic feature extraction methods. ML techniques are commonly used for handcrafted feature extraction, while DL is well-suited for automatic feature extraction. After a comprehensive literature review, we identified features that provide better inter-class separability. Inter-class separability refers to the measure that how two classes are distant, different, or separable from one another. The higher the inter-class separability, the easier it is for the classifier to distinguish and classify the classes. Conversely, the lower the inter-class separability, the more challenging for the classifier to distinguish between the classes, because lower inter-class separability indicates that the classes are overlapping significantly. Temporal and spectral features can be identified and extracted, revealing significant patterns within the EEG signal. Following preprocessing and channel selection, the temporal features were extracted including min, max, mean (Equation 3), variance (Equation 4), standard deviation (Equation 5) and skewness (Equation 6). The mean represented as μ, is calculated as follows:


(3) $$\begin{array}{l} \mu =\frac{1}{K}\overset{K}{\underset{i=1}{\sum }}\left(x_{i}\right) \end{array}$$

(4) $$\begin{array}{l} \sigma^{2}=\frac{1}{K}\overset{K}{\underset{i=1}{\sum }}{\left(x_{i}-\mu \right)}^{2} \end{array}$$

(5) $$\begin{array}{l} \sigma =\sqrt{\frac{1}{K}\overset{K}{\underset{i=1}{\sum }}{\left(x_{i}-\mu \right)}^{2}} \end{array}$$

(6) $$\begin{array}{l} S=\frac{1}{K}\overset{K}{\underset{i=1}{\sum }}{\left(x_{i}-\mu \right)}^{3} \end{array}$$

where, μ is EEG signal mean, *x*_*i*_ is value of the EEG signal at *i*^*th*^ sample, *K* is number of samples in EEG signals. Variance is the measurement value used to show how far a set of numbers is spread with respect to the mean or average value. σ^2^ is variance of EEG signals. Standard deviation is a measure representing the amount of how much dispersed or variation, such as spread, dispersion is in the data from the mean. σ is the standard deviation of EEG signals. Skewness is a measure of asymmetry of the distribution around the mean. It shows in which direction the data is skewed.

The spectral analysis of EEG signals is commonly done by obtaining the PSD. PSD is a Fourier transform of the autocorrelation function (Equation 7). PSD and auto-correlation are very closely related to each other in the analysis of signals and time series. The auto-correlation function can be calculated as:


(7) $$\begin{array}{l} R_{x}\left(\tau \right)=E\left[x\left(t\right).x\left(t+\tau \right)\right] \end{array}$$

where, *x*(*t*) is EEG signal sample, *E* is expected or mean value.

PSD describes the distribution of power over frequency and may be computed with the Fourier transform or the distribution of mean power of a signal in the frequency domain (26). The PSD is calculated as:


(8) $$\begin{array}{l} S_{x}\left(t\right)=\int_{-\infty }^{\infty }R_{x}\left(\tau \right).e^{-2\pi if\tau }df \end{array}$$

Spectral features are frequency domain features, that include spectral centroid, variational coefficient, and spectral skewness. These features can be computed with the help of PSD, which is computed by Equation 8. where, *R*_*x*_(τ) denotes autocorrelation of the signal *x*(*t*). Spectral centroid, variational coefficient, and spectral skewness can be computed by following equations.


(9) $$\begin{array}{l} C_{s}=\frac{\sum_{t}tS_{x}\left(t\right)}{\sum_{t}S_{x}\left(t\right)} \end{array}$$

(10) $$\begin{array}{l} \sigma_{s}^{2}=\frac{\sum_{t}{\left(t-C_{s}\right)}^{2}S_{x}\left(t\right)}{\sum_{t}S_{x}\left(t\right)} \end{array}$$

(11) $$\begin{array}{l} \beta_{s}=\frac{\sum_{t}{\left(\left(t-C_{s}\right)/\sigma_{s}\right)}^{3}S_{x}\left(t\right)}{\sum_{t}S_{x}\left(t\right)} \end{array}$$

Tables 1, 2 present the statistical and spectral features extracted from 10 EEG segments corresponding to the preictal and interictal states, respectively. Each table lists features such as minimum, maximum, mean, variance, standard deviation, skewness, spectral centroid, spectral variance, and spectral skewness for each segment. This layout allows for segment-wise analysis of feature variation within each class and supports comparative evaluation between preictal and interictal brain states, offering valuable insights into the distinguishing characteristics relevant for seizure prediction.

**Table 1:** Statistical and spectral features extracted from 10 EEG segments of preictal state.

**Feature**	**Seg1**	**Seg2**	**Seg3**	**Seg4**	**Seg5**	**Seg6**	**Seg7**	**Seg8**	**Seg9**	**Seg10**
Min	-0.00013	-0.00013	-0.00025	-0.00016	-9.75E-05	-0.00010	-8.15E-05	-7.33E-05	-0.00017	-0.00012
Max	0.00010	0.00013	0.00022	8.22E-05	9.20E-05	8.46E-05	6.58E-05	7.29E-05	9.24E-05	0.00014
Mean	-7.13E-08	1.20E-06	-1.16E-06	5.84E-07	1.38E-07	4.44E-07	-4.14E-07	5.12E-07	-4.70E-08	9.59E-07
Variance	9.17E-10	1.09E-09	3.57E-09	6.31E-10	6.65E-10	5.29E-10	3.81E-10	4.52E-10	8.28E-10	1.26E-09
Standard deviation	3.03E-05	3.30E-05	5.98E-05	2.51E-05	2.58E-05	2.30E-05	1.95E-05	2.13E-05	2.88E-05	3.56E-05
Skewness	-0.191	-0.166	-0.198	-1.230	-0.269	-0.271	-0.162	-0.182	-1.007	0.120
Spectral centroid	5.794	5.090	7.621	5.550	5.365	6.426	7.066	6.591	4.529	4.653
Spectral variance	36.896	45.557	293.917	55.709	47.305	58.932	67.889	59.246	38.331	32.890
Spectral skewness	4.079	6.505	4.177	5.755	5.441	4.426	4.580	3.972	5.160	4.747

**Table 2:** Statistical and spectral features extracted from 10 EEG segments of interictal state.

**Feature**	**Seg1**	**Seg2**	**Seg3**	**Seg4**	**Seg5**	**Seg6**	**Seg7**	**Seg8**	**Seg9**	**Seg10**
Min	-0.00062	-0.00083	-0.00075	-0.00075	-0.00046	-0.00015	-0.000078	-0.00063	-0.00062	-0.00070
Max	0.00074	0.00084	0.00080	0.00064	0.00065	0.00011	0.000099	0.00058	0.00062	0.00069
Mean	-1.98E-07	3.82E-07	2.00E-06	-1.94E-08	-1.29E-06	1.07E-06	1.19E-07	-8.82E-08	-3.99E-07	-5.19E-07
Variance	1.17E-08	2.06E-08	3.38E-08	2.30E-08	1.02E-08	6.95E-10	5.15E-10	7.89E-09	1.79E-08	1.56E-08
Standard deviation	1.08E-04	1.44E-04	1.84E-04	1.52E-04	1.01E-04	2.64E-05	2.27E-05	8.88E-05	1.34E-04	1.25E-04
Skewness	0.966	-0.168	0.359	-0.135	0.663	-0.599	0.283	-0.238	0.433	0.108
Spectral centroid	20.206	22.993	12.441	15.491	6.892	11.051	9.771	18.497	15.589	19.211
Spectral variance	478.771	509.770	358.245	442.970	248.950	393.654	265.381	458.774	447.203	477.597
Spectral skewness	1.527	1.447	2.345	2.012	4.128	2.770	2.881	1.663	2.019	1.718

#### 2.2.2 Customized 1DCNN for automated feature extraction

CNN is extensively utilized for EEG feature extraction and classification tasks due to its ability to automatically learn spatial patterns within the data. For automated features, we implemented 1DCNN following the preprocessing of EEG signals, which includes channel selection and data segmentation. Our proposed 1DCNN is composed of several distinct layers, designed to apply filters that identify essential patterns within the EEG signal. These layers are followed by activation functions and pooling layers. The activation function adds non-linearity to the network, which allows the network to learn complex patterns and relationships within the data and can highly reduce the dimensionality while keeping the critical information. The output of the extracted features was flattened and passed through fully connected layers for classification of interictal and preictal states. The feature-level fusion of handcrafted and automated features was also performed before passing them to the dense layer.

**Figure 8:**
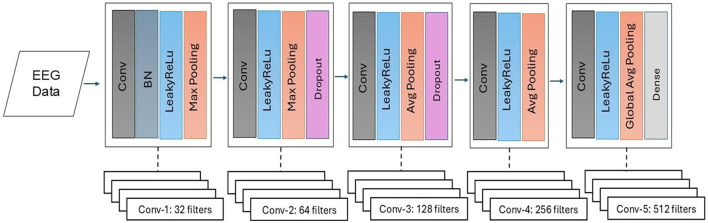
Proposed customized architecture of 1DCNN.

**Figure 9:**
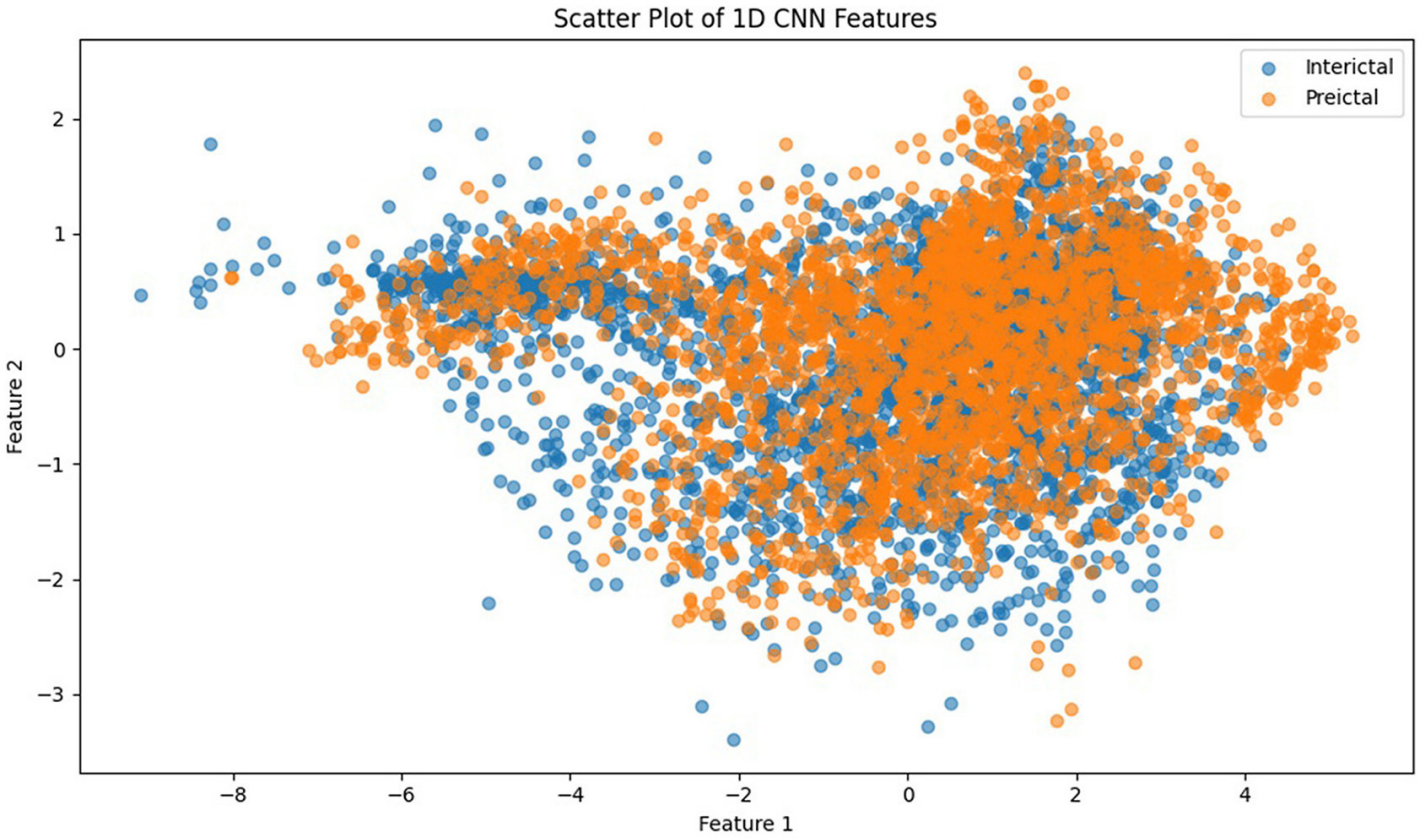
Scatter plot of 1DCNN features showing the distribution of interictal and preictal EEG segments.

Figure 8 presents the visual description of the proposed architecture of customized 1DCNN, whereas, detailed list of parameters is listed in Table 3. It begins with a Conv1D layer featuring 32 filters of size 3, followed by batch normalization and Leaky ReLu activation to stabilize the training and add non-linearity. After that MaxPool1D layer is added for down-sampling. The network succeeded with several additional convolutional layers: 64 filters of size 3, 128 and 256 filters of size 3, each followed by ReLu activation. Average pooling is applied after the third and fourth convolutional layers to reduce dimensionality with 0.5 dropout layers to mitigate overfitting. The final convolutional layer uses 512 filters, followed by a one-dimensional global average pooling layer that aggregates the features. The architecture concludes with a dense layer with an ensemble classifier for binary classification. The total number of trainable parameters in this CNN architecture is 524,449. Figure 9 illustrates the distribution of interictal and preictal EEG segments based on 1DCNN-extracted features.

**Table 3:** Proposed architecture of 1DCNN with list of parameters.

**Layer type**	**Output Shape**	**Parameters**
Conv1D	(None, 5,118, 32)	608
Batch normalization	(None, 5,118, 32)	128
Leaky ReLU	(None, 5,118, 32)	0
Max pooling 1D	(None, 2,559, 32)	0
Conv1D	(None, 2,557, 64)	6,208
Leaky ReLU	(None, 2,557, 64)	0
Max pooling 1D	(None, 1,278, 64)	0
Dropout	(None, 1,278, 64)	0
Conv1D	(None, 1,276, 128)	24,704
Leaky ReLU	(None, 1,276, 128)	0
Average pooling 1D	(None, 638, 128)	0
Dropout	(None, 638, 128)	0
Conv1D	(None, 636, 256)	98,560
Leaky ReLU	(None, 636, 256)	0
Average pooling 1D	(None, 318, 256)	0
Conv1D	(None, 316, 512)	393,728
Leaky ReLU	(None, 316, 512)	0
Global average pooling 1D	(None, 512)	0
Dense	(None, 1)	513

### 2.3 Classification of EEG signals

Once a comprehensive feature vector is extracted, preictal and interictal class samples are then classified. Given the complex nature of EEG signals and subtle differences between seizure states, relying on a single classifier can limit predictive performance. Hence, we propose an ensemble approach combining diverse classifiers (SVM, RF, and LSTM) through a meta-learning strategy to enhance prediction robustness and generalizability. We propose a novel ensemble meta learner classifier with base classifiers including SVM, RF, and LSTM to perform classification between preictal and interictal classes. We used a radial basis function (RBF) kernel in SVM due to the non-linear data, which was selected empirically. Similarly, in the case of RF, we selected 150 trees after experimentation. In case of LSTM, 32 repeating units were used, followed by meta learning classifier described in Algorithm 1.

**Algorithm 1:** Meta-learner ensemble classifier.

**Require:** Training dataset $D={\left\{\left(x_{i},y_{i}\right)\right\}}_{i=1}^{n}$, base classifiers {*C*_1_, *C*_2_, …, *C*_*m*_}, meta-learner *M*
**Ensure:** Final prediction ŷ
1: Split *D* into *D*_train_ and *D*_meta_ for training base classifiers and meta-learner respectively.
2: **for** each base classifier *C*_*k*_ in {*C*_1_, *C*_2_, …, *C*_*m*_} **do**
3: Train *C*_*k*_ on *D*_train_
4: **end for**
5: Initialize meta-training dataset *D*_meta_train_←∅
6: **for** each (*x*_*j*_, *y*_*j*_) in *D*_meta_ **do**
7: Obtain predictions {*p*_1_, *p*_2_, …, *p*_*m*_} from {*C*_1_, *C*_2_, …, *C*_*m*_} on *x*_*j*_
8: Form meta-instance *z*_*j*_ = [*p*_1_, *p*_2_, …, *p*_*m*_]
9: Add (*z*_*j*_, *y*_*j*_) to *D*_meta_train_
10: **end for**
11: Train meta-learner *M* on *D*_meta_train_
12: **Prediction Phase:**
13: Given a new instance *x*:
14: Obtain predictions {*p*_1_, *p*_2_, …, *p*_*m*_} from {*C*_1_, *C*_2_, …, *C*_*m*_} on *x*
15: Form meta-instance *z* = [*p*_1_, *p*_2_, …, *p*_*m*_]
16: Use *M* to predict ŷ from *z*
17: **return** ŷ

## 3 Results and discussion

**Figure 10:**
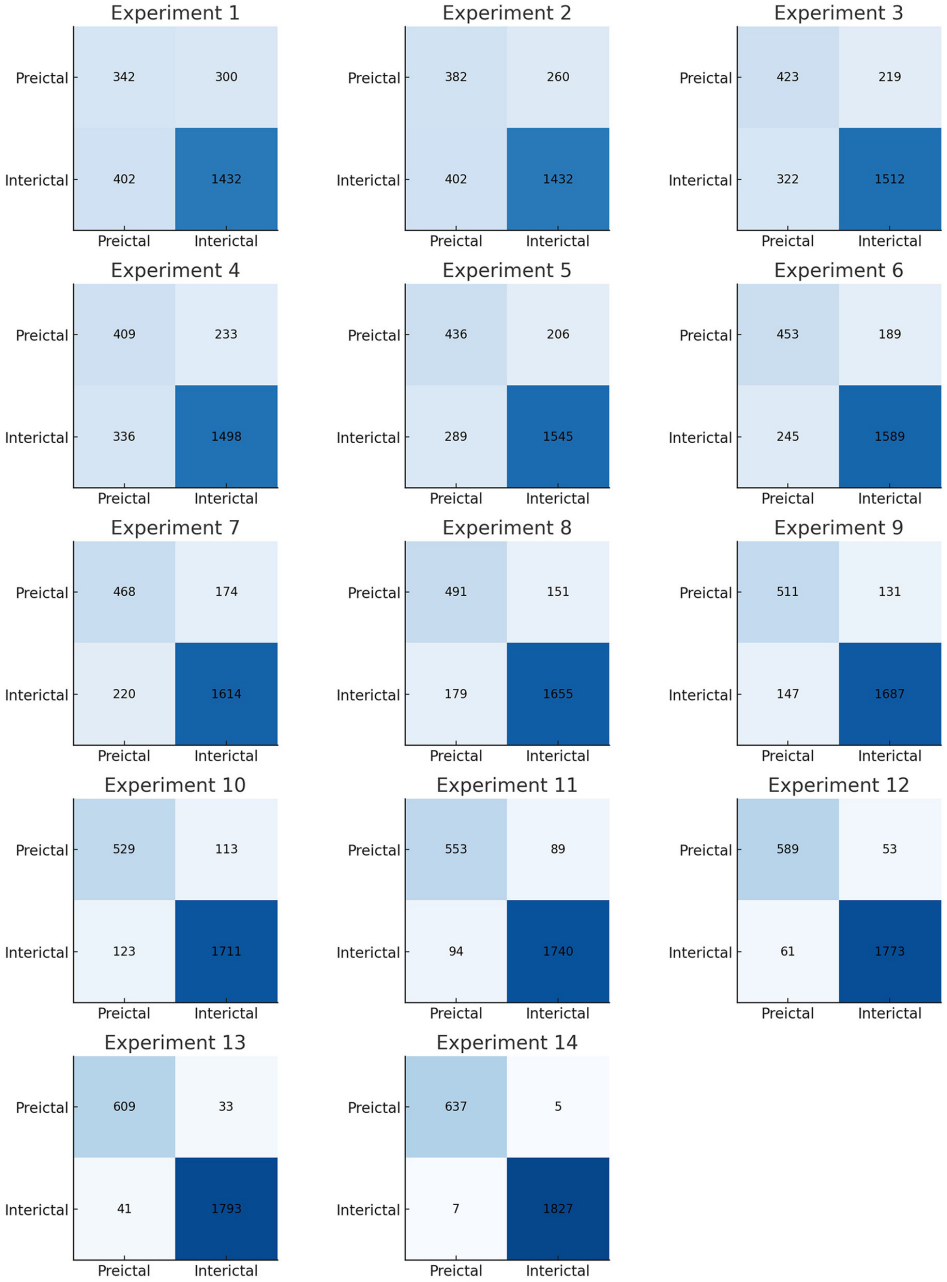
Confusion matrices of all experiments performed.

We performed multiple experiments on the CHB-MIT dataset and evaluated the methods based on accuracy, sensitivity, and specificity. Python 3 and MATLAB were used on a Windows 11 system for the implementation. The experiments for epileptic seizure prediction are performed on NVIDIA GeForce RTX 3,090 and 64 GB of RAM. All the implementations were done using Tensorflow and Scikit-learn for seizure classification. Table 4 presents the results of the ablation study performed. Figure 10 presents the confusion matrices of all experiments. We performed multiple experiments by varying approaches in preprocessing, feature extraction, and classification. In the first experimental setup, we selected a non-overlapping window and extracted temporal and spectral features, and performed classification using a kNN classifier. With this experimental setup, we achieved an accuracy of 71.65%, sensitivity and specificity of 53.27% and 78.08%, respectively. Preprocessing and feature extraction were kept the same in experiments 2 and 3, whereas RF and SVM classifiers were used for classification between preictal and interictal states. SVM achieved an accuracy of 78.15% which was more than 4% increased compared to RF. Similarly, CNN and LSTM were used for classification with the same preprocessing and feature extraction, and LSTM outperformed CNN in terms of all three performance measures.

**Table 4:** Results obtained after performing an ablation study on the CHB-MIT dataset for epileptic seizure prediction.

**Preprocessing**	**Feature extraction**	**Classification**	**Accuracy (%)**	**Sensitivity (%)**	**Specificity (%)**	**MCC**	**AUC-ROC**
Non-overlapping window	Handcrafted features	KNN	71.65	53.27	78.08	0.2997	0.6568
Non-overlapping window	Handcrafted features	RF	73.26	59.50	78.08	0.3541	0.6879
Non-overlapping window	Handcrafted features	SVM	78.15	65.89	82.44	0.4618	0.7417
Non-overlapping window	Handcrafted features	CNN	77.02	63.71	81.68	0.4337	0.7269
Non-overlapping window	Handcrafted features	LSTM	80.01	67.91	84.24	0.5023	0.7608
Non-overlapping window, Butter-worth filter	Handcrafted features	SVM	82.47	70.56	86.64	0.5572	0.7860
Non-overlapping window, Butter-worth filter, Wavelet transform	Handcrafted features	SVM	84.09	72.90	88.00	0.5958	0.8032
Non-overlapping window, Butter-worth filter, Wavelet and Fourier transform	Handcrafted features	SVM	86.67	76.48	90.24	0.6581	0.8336
Non-overlapping window, Butter-worth filter, Wavelet and Fourier transform, channel selection	Handcrafted features	SVM	88.77	79.60	91.98	0.7101	0.8579
Non-overlapping window, Butter-worth filter, Wavelet and Fourier transform, channel selection	1DCNN	SVM	90.47	82.40	93.29	0.7532	0.8775
Non-overlapping window, Butter-worth filter, Wavelet and Fourier transform, surrogate channel	Handcrafted features	SVM	92.61	86.14	94.87	0.8081	0.9051
Non-overlapping window, Butter-worth filter, Wavelet and Fourier transform, surrogate channel	1DCNN	SVM	95.40	91.74	96.67	0.8806	0.9420
Non-overlapping window, Butter-worth filter, Wavelet and Fourier transform, surrogate channel	Handcrafted and 1DCNN feature fusion	SVM	97.01	94.86	97.76	0.9225	0.9621
Non-overlapping window, Butter-worth filter, Wavelet and Fourier transform, surrogate channel	Handcrafted and 1DCNN feature fusion	Ensemble classifier	**99.52**	**99.22**	**99.62**	**0.97**	**0.9970**

Bold entries represent the highest achieved results of each metric.

Effective preprocessing plays an important role in the accurate prediction of epileptic seizures using EEG signals. Therefore, a Butterworth bandpass filter was applied to remove noise from EEG signals, whereas feature extraction and classification were kept the same, and an increased accuracy of 84.07% was observed. In the next experiments, preprocessing was further enhanced by applying the wavelet transform along with the Butterworth filter to increase the SNR, and it resulted in increased accuracy, sensitivity, and specificity. Similarly, the Fourier transform was also applied in addition to the Butterworth filter and wavelet transform, and the results were promising.

The choice of a fixed, non-overlapping 15-s window for EEG segmentation in our study was guided by its demonstrated effectiveness in prior seizure prediction research and its suitability for real-time implementation. However, we acknowledge that such static segmentation may result in the loss of critical information, particularly near transitional states such as the onset or termination of seizures. These transitions often contain subtle but clinically significant changes that may not be fully captured within rigid window boundaries. To enhance temporal sensitivity, future extensions of this work could incorporate overlapping windows or adaptive windowing strategies that dynamically adjust based on signal characteristics such as variance, entropy, or frequency shifts. Such approaches have the potential to capture transitional dynamics more effectively, improving both the responsiveness and predictive accuracy of seizure detection systems.

To assess the computational efficiency of the proposed framework, we evaluated the complete pipeline comprising preprocessing, feature extraction, and ensemble-based classification on a high-performance system equipped with an NVIDIA GeForce RTX 3090 and 64 GB of RAM. With GPU acceleration, the average processing time per 15-s EEG segment was approximately 0.12 s. This includes Butterworth filtering, wavelet and Fourier-based feature extraction, spatial filtering, and ensemble inference. The 1DCNN module benefited significantly from GPU parallelism using PyTorch, while classical models such as RF and SVM, as well as handcrafted feature operations, were efficiently handled on the CPU. All modules were implemented using optimized scientific computing libraries, including PyTorch, SciPy, and PyWavelets. The peak memory usage remained well within the hardware limits, ensuring that the proposed approach is suitable for real-time or near real-time deployment in high-throughput clinical environments.

An important aspect in real-time seizure prediction is the time taken to classify the test sample. EEG signals have high dimensionality due to the number of channels. It is extremely important to either reduce the number of channels by performing a channel selection method or by combining all channels to form a single surrogate channel. It was observed that the surrogate channel using an optimized spatial filter outperformed channel selection. It is extremely important to extract a feature vector with high interclass variance and low intraclass variance. Therefore, we propose a customized architecture of 1DCNN that consists of five convolutional layers followed by batch normalization and max pooling. A Leaky ReLU with the value of 0.01 has been used to avoid the problem of vanishing gradients. In this research, a comprehensive feature vector is formed by concatenating the handcrafted, and features extracted using a customized 1DCNN. We also propose an ensemble classifier that uses MAML with three base classifiers, including SVM, RF, and LSTM. We used *k*-fold cross validation and were able to achieve an accuracy of 99.52% along with sensitivity of 99.22% and specificity of 99.62%, with standard deviation of 0.53, 0.61, and 0.59, respectively.

**Figure 11:**
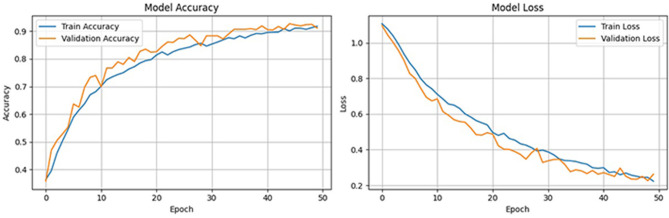
Training and validation performance curves of the proposed model over 50 epochs.

**Figure 12:**
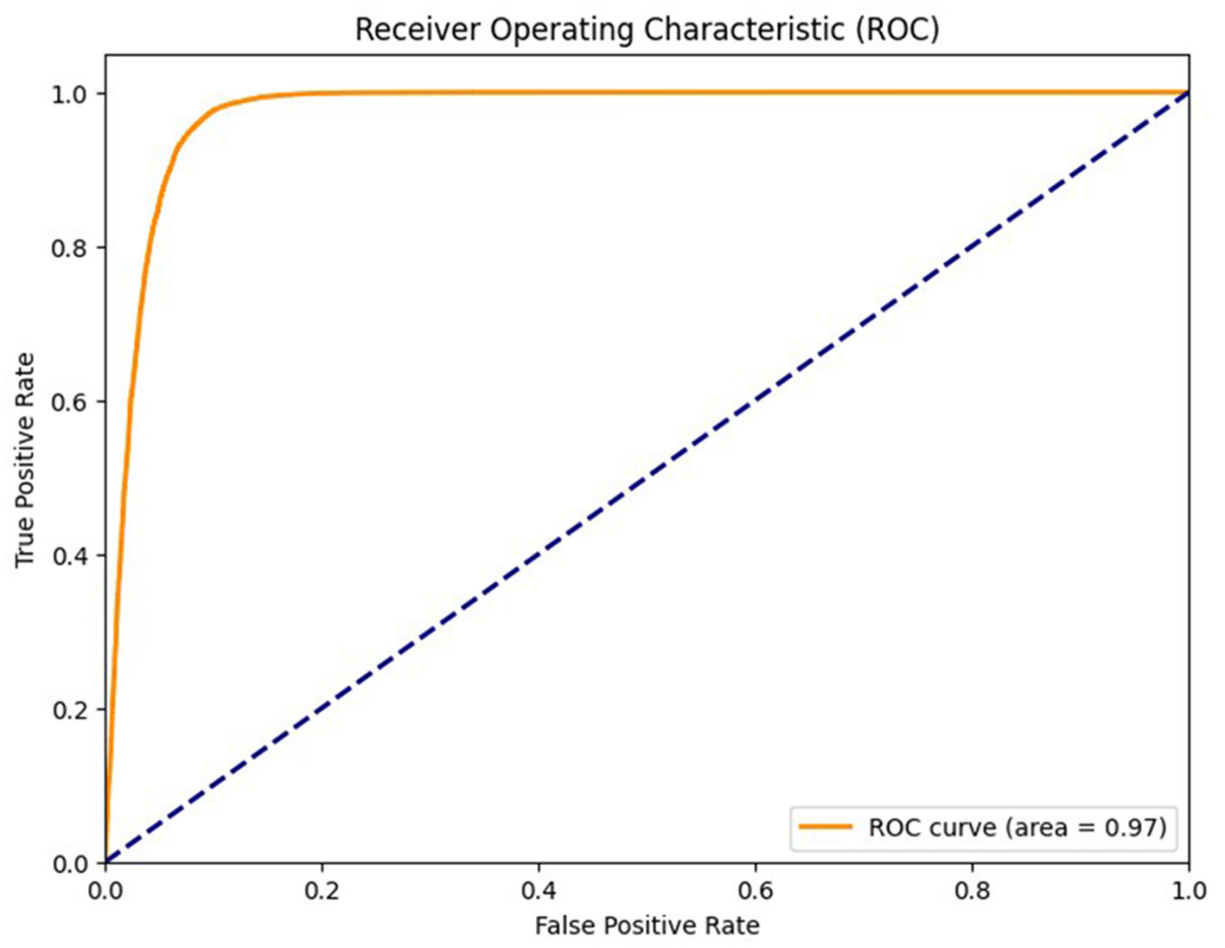
Receiver operating characteristic curve of the proposed method of epileptic seizure prediction.

To further validate the robustness of the proposed model, we computed the Matthews correlation coefficient (MCC) and the area under the receiver operating characteristic curve (AUC-ROC). The ensemble classifier achieved an MCC score of 0.99, reflecting a strong correlation between predicted and actual class labels even in the presence of class imbalance. Furthermore, the AUC-ROC score of 0.997 confirms the high discriminative power of the proposed model in distinguishing between preictal and interictal states. Figure 11 shows the ROC curve of the proposed method. To evaluate the learning behavior and check for overfitting, we plotted the training and validation accuracy and loss curves, as shown in Figure 12. Table 5 compares the performance of our proposed method with recent state-of-the-art methods proposed by researchers on the same dataset, and it shows that the proposed method outperforms not only in terms of accuracy, sensitivity, and specificity but also uses less computational power due to reduced dimensionality. Although the proposed model achieves a low false positive rate during evaluation, its practical implications must be considered in continuous monitoring scenarios. Even a few false alarms per day can lead to alarm fatigue, reduced trust in the system, and clinical inefficiencies. In real-world deployment, such issues could be mitigated by incorporating post-processing techniques such as temporal smoothing, majority voting across time windows, or hybrid decision systems that validate alerts through additional signals. These enhancements would further improve the practical viability of the proposed method in continuous, long-term monitoring contexts.

**Table 5:** Comparison of results achieved by proposed method with state-of-the-art existing methods.

**Authors**	**Accuracy (%)**	**Sensitivity (%)**	**Specificity (%)**
Birjandtalab et al. (3)	95	96.27	Not reported
Birjandtalab et al. (4)	Not reported	89.80	Not reported
Alotaiby et al. (5)	Not reported	89	37
Fei et al. (6)	89.67	89.50	89.75
Cogan et al. (7)	86	100	73
Cho et al. (8)	80.74	80.54	80.50
Jana et al. (9)	90.66	97	95.87
Daoud and Bayoumi (10)	99.60	99.72	99.6
Asharindavida et al. (11)	82.7	Not reported	Not reported
Borhade et al. (12)	96.54	96.52	97.53
Zhang et al. (13)	89.98	92.9	87.04
Usman et al. (14)	Not reported	92.7	90.8
Tamanna et al. (15)	96.38	76.73	83.16
Jana and Mukherjee (16)	99.47	97.83	92.35
Jemal et al. (17)	90.9	96.1	84.6
Koutsouvelis et al. (19)	97.32	99.31	95.34
Quadri et al. (34)	98.3	97.63	Not reported
**Proposed method**	99.47	97.83	92.35

**Figure 13:**
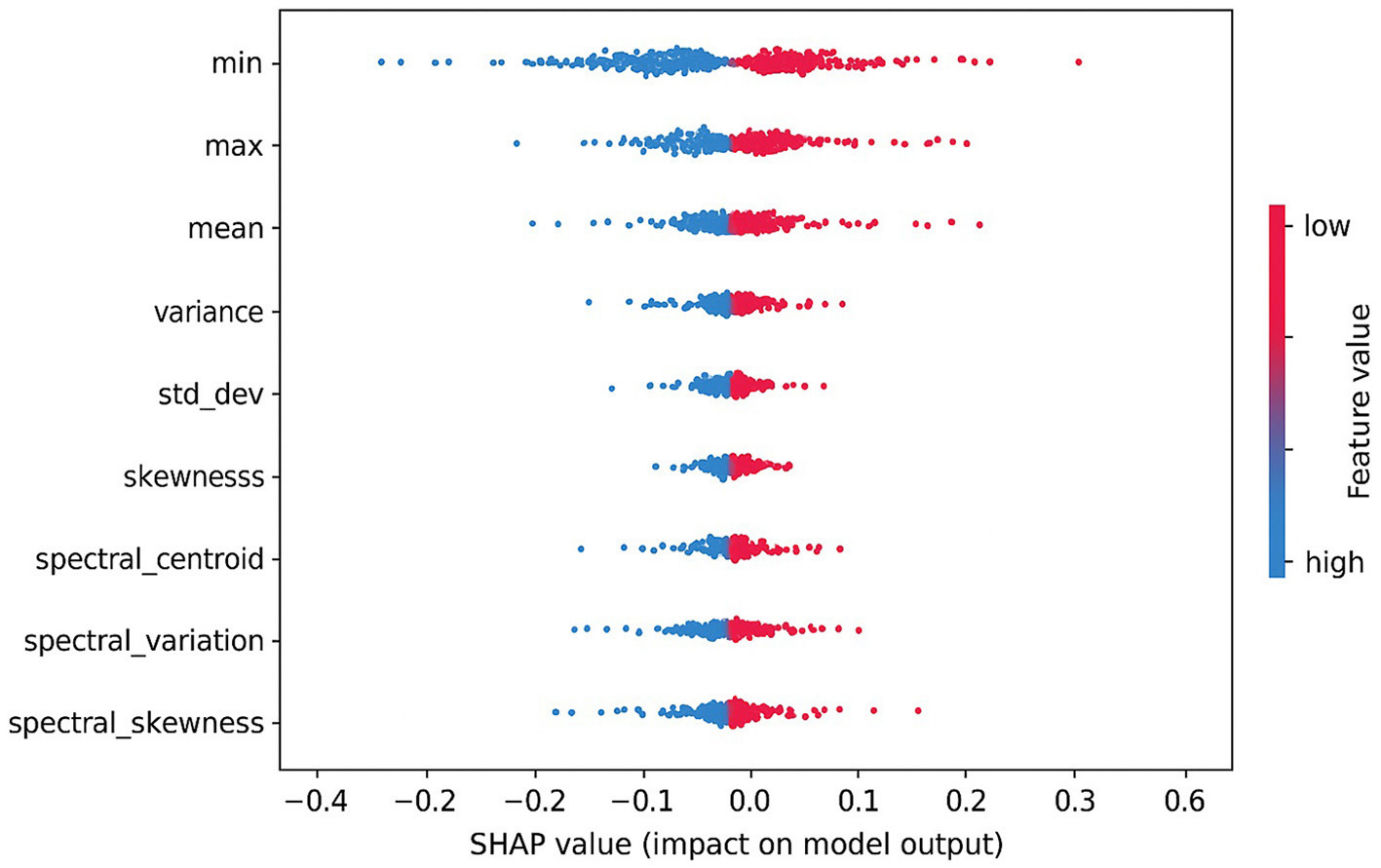
SHAP summary plot showing the impact of top handcrafted EEG features on the output of the proposed ensemble model.

To ensure transparency in model decision-making, we applied Shapley additive explanations (SHAP) to interpret the influence of individual handcrafted features on the predicted seizure class. As shown in Figure 13, features like min, max, and mean had the most significant positive impact on the model's output. The direction and magnitude of each feature's contribution can be observed from the horizontal spread of SHAP values. For instance, high values of max and mean features (indicated in red) consistently push the model toward predicting the preictal state. This interpretability analysis enhances trust in the model's outputs and provides useful insights for potential clinical validation.

## 4 Conclusion and future directions

In this research, we propose a novel method for the prediction of epileptic seizures using scalp electroencephalographic (EEG) signals. The proposed method consists of three steps, including preprocessing, feature extraction, and classification. We propose a robust preprocessing method that involves conversion of 23 channels into a single surrogate channel using an optimized spatial pattern filter to reduce the dimensionality, followed by denoising using a Butterworth filter, wavelet, and Fourier transform. We also propose a customized architecture of a one-dimensional convolutional neural network (1DCNN), which is not only lightweight but also provides a feature vector with high interclass variance. Both handcrafted and 1DCNN features are concatenated to form a feature vector, which is then fed into three classifiers, including support vector machines, random forest, long short-term memory, and a model-agnostic meta learner ensemble classifier. The proposed method performs better compared to existing state-of-the-art methods in terms of accuracy, sensitivity, and specificity, and is also computationally less complex due to reduced dimensionality and a customized light-weight architecture. In the future, integrating other physiological signals, such as heart rate and blood oxygen levels, with EEG data could provide a more comprehensive understanding of seizures before onset. The proposed method can also be applied in real-time analysis of epileptic seizures. As part of future work, we plan to develop a lightweight graphical user interface to facilitate user interaction with the proposed model. This interface will enable real-time EEG data input, feature visualization, and display of model predictions and performance metrics, thereby enhancing the practical applicability of the system in clinical or research environments.

## Data Availability

Publicly available datasets were analyzed in this study. This data can be found at: Direct Data Link: https://physionet.org/content/chbmit/1.0.0/, Repository: PhysioNet, DOI: https://doi.org/10.13026/C2K01R.
